# The influence of the way of regression on the results obtained by the receptorial responsiveness method (RRM), a procedure to estimate a change in the concentration of a pharmacological agonist near the receptor

**DOI:** 10.3389/fphar.2024.1375955

**Published:** 2024-05-02

**Authors:** Ignac Ovari, Gabor Viczjan, Tamas Erdei, Barbara Takacs, Vera Tarjanyi, Judit Zsuga, Miklos Szucs, Zoltan Szilvassy, Bela Juhasz, Rudolf Gesztelyi

**Affiliations:** ^1^ Department of Pharmacology and Pharmacotherapy, Faculty of Medicine, University of Debrecen, Debrecen, Hungary; ^2^ University of Debrecen, Doctoral School of Nutrition and Food Sciences, Debrecen, Hungary; ^3^ Department of Psychiatry, Faculty of Medicine, University of Debrecen, Debrecen, Hungary; ^4^ Department of Urology and Andrology, Kenezy Gyula Campus, University of Debrecen, Debrecen, Hungary

**Keywords:** adenosine, interstitial concentration, A1 adenosine receptor, atrium, heart, RRM, local fitting, global fitting

## Abstract

The receptorial responsiveness method (RRM) enables the estimation of a change in concentration of an (even degradable) agonist, near its receptor, *via* curve fitting to (at least) two concentration-effect (E/c) curves of a stable agonist. One curve should be generated before this change, and the other afterwards, in the same system. It follows that RRM yields a surrogate parameter (“c_x_”) as the concentration of the stable agonist being equieffective with the change in concentration of the other agonist. However, regression can be conducted several ways, which can affect the accuracy, precision and ease-of-use. This study utilized data of previous *ex vivo* investigations. Known concentrations of stable agonists were estimated with RRM by performing individual (local) or global fitting, this latter with one or two model(s), using a logarithmic (logc_x_) or a nonlogarithmic (c_x_) parameter (the latter in a complex or in a simplified equation), with ordinary least-squares or robust regression, and with an “all-at-once” or “pairwise” fitting manner. We found that the simplified model containing logc_x_ was superior to all alternative models. The most complicated individual regression was the most accurate, followed closely by the moderately complicated two-model global regression and then by the easy-to-perform one-model global regression. The two-model global fitting was the most precise, followed by the individual fitting (closely) and by the one-model global fitting (from afar). Pairwise fitting (two E/c curves at once) improved the estimation. Thus, the two-model global fitting, performed pairwise, and the individual fitting are recommended for RRM, using the simplified model containing logc_x_.

## 1 Introduction

Measuring the concentration of small molecules in biological samples is generally considered to be an issue for analytical chemistry that can now be performed to a high standard (Valcárcel, 2000; Belanger et al., 2020). However, concentration determination of small molecules with a short half-life and/or strong compartmentalization tendency in living, functioning, moreover moving tissues is still a challenge (Karsai et al., 2006; Ramakers et al., 2008; Zhang et al., 2015; Markiewicz et al., 2022). If certain requirements are met, the receptorial responsiveness method (RRM) can help to solve (or bridge) this problem. RRM can quantify an acute (and occasionally even chronic) increase in the concentration of a pharmacological agonist in the microenvironment of its receptors (Gesztelyi et al., 2004; Karsai et al., 2006). (Using this method “in reverse” (i.e., by reversing the temporality), it is of course also possible to determine a decrease in concentration.)

The principle of RRM may seem to be complicated at first glance but is in fact straightforward (the main challenge to understand it stems from its interdisciplinary nature). First, it is worth considering a thought experiment. Let us administer the same dose of an agonist twice to a biological system, and then let us compute the effect of both doses. The correct way is to define the same initial state for both effects (namely, the state before the administration of the first dose) and to take also the first dose into account when calculating the effect of the second one (because, due to its magnitude relative to the second dose, the first dose is not negligible). However, if the effect of the second dose is calculated without taking the first dose into account, and the condition after the first dose is taken as the initial state, the effect computed for the second dose will be smaller than that for the first dose (although the two doses equal).

The explanation for this smaller effect is that the agonist molecules administered with the first dose, by binding to a fraction of the receptors and then by eliciting an effect on them, consume part of the response capacity of the system. For this reason, the agonist molecules administered with the second dose will act in a system with submaximal response capacity. If, for example, the dose in question is high enough to exert a (practically) maximal effect after its first administration, no further effect can develop, thus, when administering it for the second time, the system will be unable to respond. Thus, the decrease in the responsiveness (observed this way) depends on the magnitude of the ignored dose, more precisely on the agonist concentration near the relevant receptors. This phenomenon can be exploited to quantify an unknown concentration of an agonist developed in the tissue compartment of the binding site of the specific receptors (Gesztelyi et al., 2004; Karsai et al., 2006; 2007; Grenczer et al., 2010a).

It should be emphasized that this phenomenon, although superficially reminiscent of it, is not based on receptor desensitization (where the effect is correctly computed but smaller than expected). In turn, the receptor desensitization interferes with the above-mentioned phenomenon, thus this latter can only be used for concentration estimation if the receptor in question desensitizes slowly relative to the time window of the determination (in our experiments: about 10–40 min, depending on the circumstances). The A_1_ adenosine receptor belongs to the especially slowly desensitizing receptors (Willems et al., 2006; Klaasse et al., 2008; Mundell and Kelly, 2011).

Of course, the effect of an agonist depends not only on its concentration in the vicinity of its receptors and on the genuine sensitivity of these receptors but also on numerous other factors (e.g., receptor density, receptor reserve, receptor isoforms, receptor trafficking, postreceptorial signaling). Moreover, the tissue concentration of the given agonist is also influenced by several factors (such as stability of the agonist including its resistance to tissue enzymes, carriers and possibly reactive endogenous compounds) (for a review, see: Kenakin and Pharmacology primer, 2022). However, when comparing two states of a system (in our case, to assess a decrease in the responsiveness of the system), the non-varying system characteristics can be cancelled so as not to disturb the determination of a single cause of the difference (which, in our case, is the absence or presence of a disregarded agonist concentration). This is a kind of application of the pharmacological null method (or null analysis), which is a useful procedure to negate cell- and tissue-dependent characteristics irrelevant for the given investigation. Nevertheless, the cost of this advantage is that measurements based on the null method require the permanence of the biological system throughout the investigation (Kenakin and Pharmacology primer, 2022).

The phenomenon described above can also be observed if the agonist being present first and being disregarded is different from that added subsequently, but they both evoke the same response (so the same response capacity is consumed by them). To do so, it is enough if they are agonists on the same receptor, or even if they influence the same postreceptorial signaling in the same direction (although binding to different receptors). So, the essence of the phenomenon is the bias in the measurement that is caused by the neglect of the first agonist. Thus, the first and disregarded agonist can be named as “biasing” or “distorting” agonist (Gesztelyi et al., 2004; Grenczer et al., 2010a).

It should be noted that the terms “bias” and “biasing” mentioned above are not related to the emerging concept of biased agonism. According to that concept, a biased (also called functionally selective) agonist is the one that does not activate every signaling pathway linked to the given receptor, but it does stimulate at least one of them. The phenomenon of biased agonism has extensively been observed for G protein-coupled receptors (Jarpe et al., 1998; Andresen, 2011; Maguire, 2016; Kenakin, 2024), including the A_1_ adenosine receptor (Franco et al., 2021; McNeill et al., 2021; IJzerman et al., 2022). Thus, to avoid misunderstanding, the agonist first present and disregarded will be called as distorting (and not biasing) agonist, and its concentration will be referred to as distorting concentration throughout this paper. Again, the term “distortion” used herein means an inevitable modification of the measured data and the consequent transformation of the curves generated from these data, under well-circumscribed conditions (see above).

**FIGURE 1:**
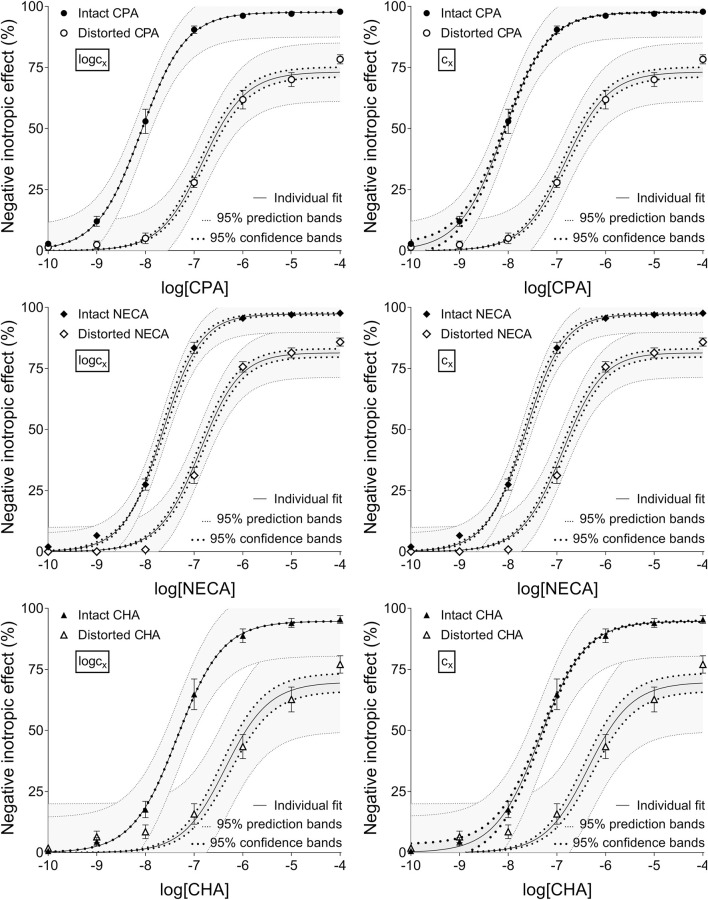
The implementation of RRM with individual regression combined with ordinary least-squares fitting, using two equations: the Eq. 8 that contained the common logarithm of the concentration to be determined (logc_x_; left panels), and the Eq. 7 containing this concentration itself (c_x_; right panels), as a parameter. The measurement was performed on “Intact”–“Distorted” concentration-effect (E/c) curve pairs obtained from a previous *ex vivo* study (Szabo et al., 2019a). The x-axis shows the common logarithm of the molar concentration of one of three synthetic A_1_ adenosine receptor full agonists (CPA, NECA, CHA) that was administered for the construction of the E/c curves. The y-axis shows the effect (expressed as the percentage decrease in the initial contractile force of isolated, paced guinea pig left atria). The “Intact” E/c curves (filled symbols) were generated in a conventional way, whereas the “Distorted” E/c curves (open symbols) were constructed in the presence of a single, disregarded extra concentration of the agonist used for the E/c curve. The symbols indicate the responses to the agonists averaged within the groups (±SEM). The continuous lines show the best-fit curves of the fitted Eqs 7, 8 (encompassing c_x_ or logc_x_, respectively). The thick dotted lines indicate the 95% confidence bands, while the thin dotted lines denote the 95% prediction bands. RRM: receptorial responsiveness method; CPA: *N* ^*6*^-cyclopentyladenosine; NECA: 5′-(N-ethylcarboxamido) adenosine; CHA: *N* ^*6*^-cyclohexyladenosine.

**FIGURE 2:**
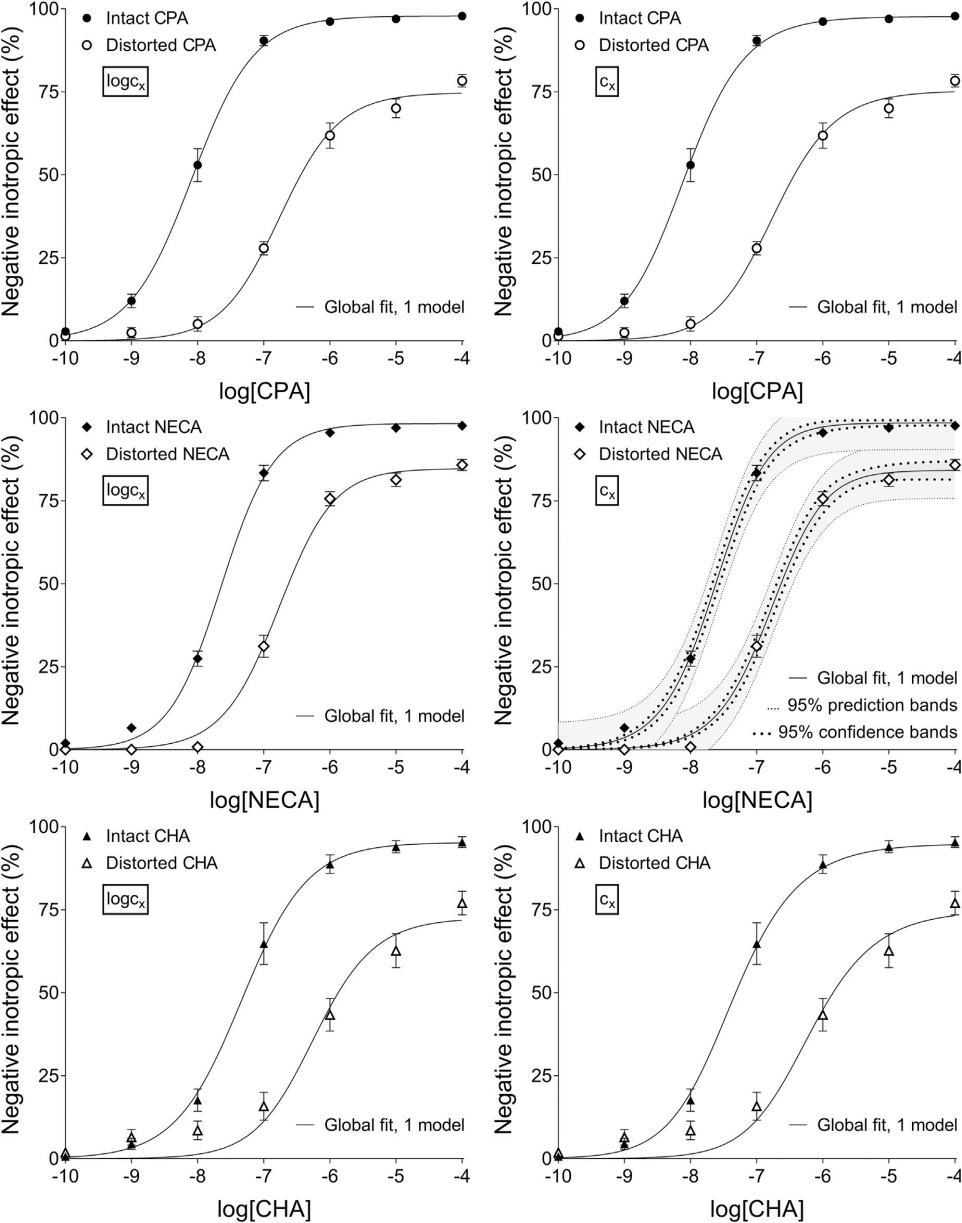
The application of RRM with one-model global regression combined with ordinary least-squares fitting, using two equations: the Eq. 8 containing the common logarithm of the concentration to be determined (logc_x_; left panels), and the Eq. 7 including this concentration itself (c_x_; right panels), as a parameter. This procedure was performed on “Intact”–“Distorted” concentration-effect (E/c) curve pairs obtained from an earlier *ex vivo* study (Szabo et al., 2019a). The x-axis shows the common logarithm of the molar concentration of one of three synthetic A_1_ adenosine receptor full agonists (CPA, NECA, CHA) that was administered for generating the E/c curves. The y-axis indicates the effect (the percentage decrease in the initial contractile force of isolated, paced guinea pig left atria). The “Intact” E/c curves (filled symbols) were constructed conventionally, whereas the “Distorted” E/c curves (open symbols) were generated in the presence of a single, disregarded surplus concentration of the agonist used for the E/c curve. The symbols indicate the responses to the agonists averaged within the groups (±SEM). The continuous lines show the best-fit curves of the fitted Eqs 7, 8. The thick dotted lines indicate the 95% confidence bands, while the thin dotted lines denote the 95% prediction bands (if any). RRM: receptorial responsiveness method; CPA: *N* ^*6*^-cyclopentyladenosine; NECA: 5′-(N-ethylcarboxamido)adenosine; CHA: *N* ^*6*^-cyclohexyladenosine.

**FIGURE 3:**
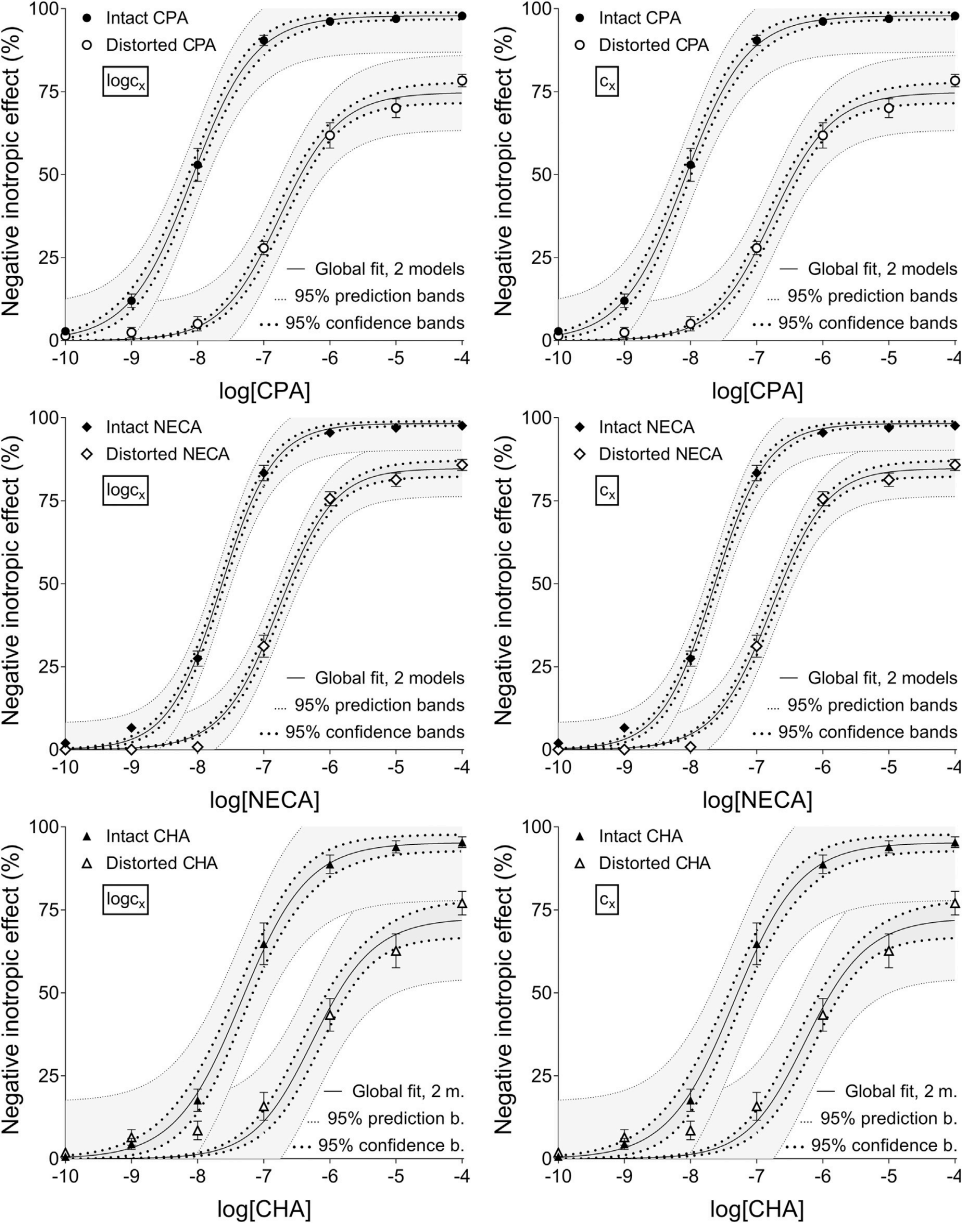
The implementation of RRM with two-model global regression combined with ordinary least-squares fitting, using two equations (as alternatives in addition to the Eq. 2 representing the Hill model): the Eq. 8 that contained the common logarithm of the concentration to be determined (logc_x_; left panels), and the Eq. 7 containing this concentration itself (c_x_; right panels), as a parameter. This procedure was performed on “Intact”–“Distorted” concentration-effect (E/c) curve pairs obtained from a previous *ex vivo* study (Szabo et al., 2019a). The x-axis shows the common logarithm of the molar concentration of one of three synthetic A_1_ adenosine receptor full agonists (CPA, NECA, CHA) that was administered for constructing the E/c curves. The y-axis indicates the effect (the percentage decrease in the initial contractile force of isolated, paced guinea pig left atria). The “Intact” E/c curves (filled symbols) were generated in a conventional way, while the “Distorted” E/c curves (open symbols) were constructed in the presence of a single, disregarded extra concentration of the agonist used for the E/c curve. The symbols indicate the responses to the agonists averaged within the groups (±SEM). The continuous lines show the best-fit curves of the Eq 2 and the Eqs 7, 8. The Eq. 2 was fitted to the “Intact” E/c curves, whereas the Eq. 8 (left panels) or 7 (right panels) was fitted to the “Distorted” ones. The thick dotted lines indicate the 95% confidence bands, while the thin dotted lines represent the 95% prediction bands. RRM: receptorial responsiveness method; CPA: *N* ^*6*^-cyclopentyladenosine; NECA: 5′-(N-ethylcarboxamido)adenosine; CHA: *N* ^*6*^-cyclohexyladenosine

It is worth noting here that NECA, CPA and CHA, three high-efficacy A_1_ adenosine receptor agonists responsible for data evaluated in the present investigation (for more detail, see legends of Figures 1–3), activate identical postreceptorial signaling pathways. However, their activation patterns slightly differ, indicating some level of functional selectivity for these agonists (Verzijl and Ijzerman, 2011). Despite their differences, NECA, CPA and CHA all have relatively long half-life in the living tissues (Pavan and IJzerman, 1998; Fredholm et al., 2001), ensuring the permanence of the experimental system that is needed for RRM (Gesztelyi et al., 2004; Karsai et al., 2006).

A complete concentration-effect (E/c) curve (ranging from ineffective to saturative concentrations) encompasses the information needed for this concentration estimation with high redundancy, and thereby offers the possibility of a more reliable estimation than what a single concentration - effect pair could provide. Consequently, the best way to assess a change (“distortion”) in a biological response is to analyze E/c curves. Two types of E/c curves are required: one holding information on the real (intact) relationship between agonist concentration and effect in the given system (serving as a benchmark), and another one informing about the extent of the distortion (and thereby, in our case, about the magnitude of the distorting concentration). These two E/c curves can be referred to as “intact” and “distorted” that contain “intact” and “distorted” effects, respectively. The intact effect and the corresponding distorted effect are assigned to the same agonist concentration, the only difference between them is the presence and neglect of a distorting concentration in the case of the distorted effect (Gesztelyi et al., 2004; Grenczer et al., 2010a). (It is worth anticipating that if more than one distorting concentration is to be estimated in the same system, then more than two E/c curves are to be assessed with RRM. This situation can technically be handled in two different ways, see further on).

The essence of RRM is to fit its model to a distorted E/c curve, utilizing the relevant pieces of information implied in the corresponding intact E/c curve. The model of RRM is a fusion of two equations: **1**) the basic equation of RRM (see later) that describes the relationship between an intact effect and its distorted counterpart, and **2**) a quantitative receptor function model (that is generally the Hill equation: Gesztelyi et al., 2012a). The information in the intact E/c curve can be made accessible two main ways. On one hand, the given receptor function model can be fitted to the intact E/c curve, and then the identical parameters in the RRM’s model can be constrained at the obtained values. After this “individualization”, the RRM’s model can simply be fitted to the corresponding distorted E/c curve (Gesztelyi et al., 2004; Grenczer et al., 2010a; 2010b). In this paper (similarly to the recent ones: Szabo et al., 2019a; Viczjan et al., 2021; 2022), this procedure will be referred to as individual regression. (Alternatively, this type of fitting can be called local regression, as opposed to global regression: Anjos et al., 2020; Spatial Data Science, 2024). On the other hand, the intact and distorted E/c curves can be fitted at once, *via* global regression. Global regression can also be performed in two ways: fitting the intact and distorted E/c curves to one model (which is the model of RRM: Szabo et al., 2019a), or fitting these E/c curves to two models (the selected receptor function model and the compatible RRM’s model). This latter maneuver should be done so that the receptor function model fits only the intact E/c curve and the RRM’s model fits exclusively the distorted E/c curve, with the common parameters shared between the two models (Curve Fitting Guide, 2024). Hereinafter, these two procedures will be referred to as one-model global regression and two-model global regression, respectively.

Finally, RRM provides a best-fit value as a measure for the distorting concentration. This best-fit value can be a concentration (c_x_) or its common logarithm (logc_x_), depending on which parameter (c_x_ or logc_x_) is included in the RRM’s model used. So, the estimate yielded by RRM is the best-fit value, if it is c_x_, or the antilog of the best-fit value, if this latter is logc_x_. The nature of this estimate depends on the fact whether the distorting agonist and the agonist used for the E/c curves are the same or not. If so, the estimate directly applies to the distorting concentration. If not, then the estimate is the concentration of the agonist used for the E/c curves that is equieffective with the distorting concentration (Gesztelyi et al., 2004; Grenczer et al., 2010a). Although this latter estimate is just a surrogate, it has the advantage that a stable agonist can be used to determine the distorting concentration of an agonist that may be degradable and/or exhibit strong compartmentalization. Importantly, since the estimate provided by RRM is derived from E/c curves, it applies to the (average) concentration of the distorting agonist near the receptors involved in the production of the effect measured (Gesztelyi et al., 2004; Karsai et al., 2006; 2007; Viczjan et al., 2022).

In a previous study (Szabo et al., 2019a), estimates of RRM were found to be more accurate and precise when obtained from individual regression rather than one-model global fitting. This may be surprising in light of that global regression is thought to be more powerful and reliable than the individual one, especially in the case of limited data and/or data with much scatter (Knutson et al., 1983; Motulsky and Christopoulos, 2004; Herman and Lee, 2012). The worse than expected performance of the one-model global fitting was then attributed to the lack of accurate estimates for the intact E/c curves, because, in that study (Szabo et al., 2019a), the model of RRM contained logc_x_ as a parameter (instead of c_x_). Hence, RRM could not yield zero, the correct estimate for the intact E/c curves, as the logarithm of zero is not defined.

Earlier computer programs calculated only symmetrical confidence intervals (CIs). Therefore, quantities typically following a log-normal distribution (e.g., concentrations) were recommended to be used as common logarithms in the equations to fit, in order to get correct CIs (Motulsky and Christopoulos, 2004). If asymmetry of CIs can be handled (not uncommon for modern statistical software systems), the use of concentrations as a logarithm in the regression models seems to be less important.

Consequently, in the present study, our first goal was to explore whether the model of RRM containing c_x_ is superior to that including logc_x_ (during one-model global regression). As an alternative approach to the issue of logc_x_ as a fitted parameter, we also investigated whether the two-model global regression, another way to avoid fitting logc_x_ to an intact E/c curve, can provide better results than the one-model global regression and perhaps than the individual fitting. Furthermore, we aimed to find out whether reducing the complexity of the model of RRM improves the results of RRM. In this context, the influence of dividing by c_x_ in the RRM’s model (to simplify its expression) had to be considered as well. In addition, regarding their impact on the outcome of RRM, the distribution of the scatter of E/c curve data and the fitting to E/c curve families (where more than one distorted E/c curve belongs to one intact E/c curve) were also addressed.

## 2 Materials and methods

### 2.1 Data analyzed

Investigations of the present study were carried out on cumulative, graded E/c curves, originally published in two earlier studies from our lab (Gesztelyi et al., 2004; Szabo et al., 2019a). The E/c curves were constructed with A_1_ adenosine receptor agonists, by measuring the contractile force of isolated, paced guinea pig left atria. These E/c curves were E/c curve pairs or families (one intact E/c curve with one or more than one related distorted E/c curve(s), respectively). These E/c curves were reevaluated with RRM implemented by combining several ways of regression, in order to compare the usefulness of the different possibilities. The intact - distorted E/c curve pairs (obtained from Szabo et al., 2019a) were reexamined by combining the following options: **1**) the RRM’s model containing logc_x_ vs*.* c_x_; **2**) the originally developed (Gesztelyi et al., 2004), more complex RRM’s model (containing c_x_) vs*.* an RRM’s model simplified with a division by c_x_ (containing c_x_ or logc_x_); **3**) individual regression vs*.* one-model global regression vs*.* two-model global regression; and **4**) ordinary least-squares (simply: ordinary) fitting vs*.* robust fitting. To reevaluate the E/c curve families (obtained from Gesztelyi et al., 2004), the following options were combined: **1**) individual fitting vs*.* one-model global fitting vs*.* two-model global fitting; **2**) ordinary regression vs*.* robust regression; and **3**) fitting all related E/c curves at once (i.e., all-at-once manner) vs*.* fitting the intact E/c curve together with only one related distorted E/c curve at once (i.e., pairwise manner).

The intact E/c curves were generated with CPA, NECA or CHA, stable, synthetic A_1_ adenosine receptor agonists with long half-life (for more detail, see legends of Figures 1–3), in the absence of any previously added adenosine receptor agonist. The distorted E/c curves were constructed with the same agonists, but in the presence of a single, known, previously administered concentration of the agonist used for the E/c curve. The distorted E/c curves were started when the effect of the surplus agonist concentration had fully developed. The surplus concentration (and its effect) was disregarded during the evaluation to make it a distorting concentration.

All E/c curves, mentioned and presented in this study, were produced from individual E/c curves (i.e., one atrium–one curve) through averaging them within the experimental groups by the curve fitting software. The averaging process preserved the individuality of the effect values (as “replicate Y values”) (Curve Fitting Guide, 2024).

Each intact - distorted E/c curve pair (Szabo et al., 2019a) held information about one distorting concentration near to the EC_50_ of the given agonist, while each intact - distorted E/c curve family (Gesztelyi et al., 2004) informed about three distorting concentrations spanning two orders of magnitude including the EC_50_ of the agonist used.

### 2.2 Regression manners dealing with models, variable parameters and sharing

To characterize the intact E/c relationship, the Hill model was chosen (Gesztelyi et al., 2012a):

$$E=E_{\max}∙\frac{c^{n}}{c^{n}+{{EC}_{50}}^{n}}=\frac{E_{\max}}{1+{\left(\frac{{EC}_{50}}{c}\right)}^{n}}$$ (1)

To fit the intact E/c curves, instead of the classical form of the Hill equation (the left-hand expression of the Eq 1), a simplified version (the right-hand expression of the Eq 1) was used, in a form where all concentrations (c and EC_50_) were expressed as a logarithm (10^logc^ and 10^logEC50^, respectively):

$$E=\frac{E_{\max}}{1+10^{n\cdot \left(\log EC_{50}-\log c\right)}}$$ (2)

where: c: the concentration of the agonist that was administered during the construction of the E/c curve; E: the effect of c (considered to be intact because of the lack of any distorting factor); E_max_: the maximal effect (achievable with the given agonist in the system in question); EC_50_: the agonist concentration producing half-maximal effect; n: the Hill coefficient (slope factor). During curve fitting, in every case, logc was the independent variable and logEC_50_ served as a parameter (see Appendix A).

To determine the distorting concentration, RRM was applied. The basic equation of RRM is as follows (Gesztelyi et al., 2004; Grenczer et al., 2010a):

$$E^{\prime }=100-\frac{100∙\left(100-E\right)}{100-E_{x}}$$ (3)

where: E’: the distorted effect that was calculated as if it had exclusively been the effect of c, regardless of the presence of c_x_ (c_x_, the distorting concentration, is always attributed to the same agonist as c by RRM, and in this study, c_x_ and c belonged indeed to the same agonist); E: the intact counterpart of E′, i.e., the effect that properly reflects the co-action of c and c_x_; E_x_: the effect of c_x_ alone (that is also intact, similarly to E).

The model of RRM was derived from the fusion of Eqs 1, 3 (a procedure similar to the combination of the Hill model and the Schild equation: Waud et al., 1978; Motulsky and Christopoulos, 2004; Gesztelyi et al., 2012b). The model of RRM can be expressed in several, algebraically equivalent forms, five of which, relevant for the present investigation, are as follows:

$$E^{\prime }=100-\frac{100\cdot \left(100-E_{\max}∙\frac{{\left(c_{x}+c\right)}^{n}}{{\left(c_{x}+c\right)}^{n}+{{EC}_{50}}^{n}}\right)}{100-E_{\max}∙\frac{{c_{x}}^{n}}{{c_{x}}^{n}+{{EC}_{50}}^{n}}}$$ (4)

$$E^{\prime }=100-\frac{100\cdot \left(100-\frac{E_{\max}}{1+{\left(\frac{{EC}_{50}}{c_{x}+c}\right)}^{n}}\right)}{100-\frac{E_{\max}}{1+{\left(\frac{{EC}_{50}}{c_{x}}\right)}^{n}}}$$ (5)

$$E^{\prime }=100-\frac{100\cdot \left(100-E_{\max}∙\frac{{\left(c_{x}+10^{logc}\right)}^{n}}{{\left(c_{x}+10^{logc}\right)}^{n}+10^{n∙\log{EC}_{50}}}\right)}{100-E_{\max}∙\frac{{c_{x}}^{n}}{{c_{x}}^{n}+10^{n∙\log{EC}_{50}}}}$$ (6)

$$E^{\prime }=100-\frac{100\cdot \left(100-\frac{E_{\max}}{1+10^{n\cdot \left(\log EC_{50}-\log\left(c_{x}+10^{\log c}\right)\right)}}\right)}{100-\frac{E_{\max}}{1+10^{n\cdot \left(\log EC_{50}-\log\left(c_{x}\right)\right)}}}$$ (7)

$$E^{\prime }=100-\frac{100\cdot \left(100-\frac{E_{\max}}{1+10^{n\cdot \left(\log EC_{50}-\log\left(10^{\log c_{x}}+10^{\log c}\right)\right)}}\right)}{100-\frac{E_{\max}}{1+10^{n\cdot \left(\log EC_{50}-\log c_{x}\right)}}}$$ (8)

where: c: the concentration of the agonist administered during the construction of the E/c curve; c_x_: the distorting concentration; E’: the distorted effect; E_max_, logEC_50_ and n: the parameters from the Hill equation (see Eqs 1, 2).

The Eq 6 stems from the Eq 4, by expressing c and EC_50_ in logarithmic form (as 10^logc^ and 10^logEC50^). In turn, Eqs 7–8 are derived from the Eq 5, the version of the Eq 4 simplified by a division by c_x_, also by expressing c and EC_50_ in logarithmic form. While Eqs 6, 7 have retained c_x_ from the previous equations, the Eq 8 contains logc_x_ as a parameter (Table 1).

**TABLE 1:** The five algebraically equivalent forms of the model of RRM presented in this study (Eqs 4–8), and three of them used for curve fitting (Eqs 6–8), highlighted in bold. The comparison of Eqs 6, 7 provides information about the influence of the complexity of the fitted equation as well as about the effect of the division by c_x_ (as c_x_ should be allowed to be zero, theoretically it should not be used for division). In turn, the comparison of Eqs 7, 8 informs about the impact of the fitted form of the distorting concentration (c_x_ or logc_x_). c (and logc): the concentration of the agonist administered during the construction of the E/c curve (and its logarithm), the independent variable in the model of RRM; EC_50_ (and logEC_50_): the half-maximal effective concentration of the agonist administered during the construction of the E/c curve (and its logarithm), a parameter in the model of RRM; c_x_ (and logc_x_): the concentration of the distorting agonist (and its logarithm), the obligate variable parameter in the model of RRM.

RRM’s models	Use of c_x_ with		Use of logc_x_ with logc and logEC_50_
	c and EC_50_	Logc and logEC_50_	
Complex, no division by c_x_	Eq 4	**Eq 6**	
Simpler, division by c_x_	Eq 5	**Eq 7**	**Eq 8**

Thus, Eqs 6, 7 gave the sought estimate itself, whereas the Eq 8 provided the estimate after taking the antilog of its best-fit value. In Eqs 6-8, E_max_, logEC_50_ and n were parameters in addition to c_x_ or logc_x_, while logc and E’ were the independent variable and the dependent variable, respectively (see Appendix A).

It should be emphasized that, during its derivation, the Eq 6 has not undergone a division by c_x_, and it contains c_x_ as a parameter. Consequently, the Eq 6 allows, in all ways, c_x_ to be zero. In turn, the Eq 7, although it incorporates c_x_ as a parameter, stems from a division by c_x_. Thus, it can be regarded theoretically questionable whether the Eq 7 allows c_x_ to be zero. Nevertheless, the Eq 7 is simpler than the Eq 6. Eventually, the Eq 8 comes from a division by c_x_ and includes logc_x_, so it does not permit c_x_ to be zero at all (Table 1).

To fit the intact and distorted E/c curves, the Eqs 6-8 were used in three ways: individually, globally with one model, and globally with two models.

During the individual regression, one of Eqs 6-8 was fitted to one E/c curve at once (either distorted or intact), in a manner that the Hill parameters (E_max_, logEC_50_ and n) were constrained to constant values. These values were provided by a previous fitting of the Eq 2 to the corresponding (or the same) intact E/c curve. So, during individual regression, of the four parameters in Eqs 6-8, only one was variable (logc_x_ or c_x_).

During global regression, all parameters of Eqs 6-8 were variable (E_max_, logEC_50_, n and logc_x_ or c_x_). When using one model, the related intact and distorted E/c curves were simultaneously fitted to one of Eqs 6-8 with the Hill parameters shared between (or among) the curves. When using two models, the related intact and distorted E/c curves were also simultaneously fitted but in a way that the Eq 2 was fitted to the intact E/c curve, while one of Eqs 6-8 was fitted to the related distorted E/c curve(s), with Hill parameters shared between the equations as well as between (or among) the curves.

When more than one distorted E/c curve belonged to one intact E/c curve, an additional choice was available for the global regression (irrespective of the number of models): curve fitting at once to all related E/c curves (all-at-once manner), and curve fitting simultaneously to only two related E/c curves, one of which was compulsorily the intact E/c curve (pairwise manner). Both options were carried out.

During the individual and one-model global ways of regression, c_x_ or logc_x_ values were determined for the intact E/c curves as well, as an additional control (since the expected value for the estimate was zero). The two-model global fitting provided no estimates for the intact E/c curves because they were fitted only to the Hill model (Eq 2).

### 2.3 Regression manners addressing data scatter

The above-mentioned ways of regression were further combined with ordinary or robust fitting, addressing the distribution of the scatter of data points around the best-fit curve. Upon robust fitting, only accuracy (but not precision) could be compared, because robust regression prevented obtaining 95% CIs and 95% confidence and prediction bands (Curve Fitting Guide, 2024).

### 2.4 Data processing and presentation

Curve plotting and fitting was performed with GraphPad Prism 9.5.1 for Windows (GraphPad Software Inc., La Jolla, CA, USA). Some operations were made with Microsoft Excel for Microsoft 365 (Microsoft Co., Redmond, WA, USA).

Accuracy of the regression was judged by the distance of the estimate from the corresponding known distorting concentration.

Precision of the regression was characterized by the width of the 95% CI of the best-fit value (c_x_ or logc_x_) addressing the distorting concentration. In addition, precision of the curve fitting and precision of the E/c curve data were characterized by the distance of the 95% confidence and prediction bands, respectively, from the corresponding best-fit curve.

Further information supplied by a 95% CI was the position of the best-fit value within it (Curve Fitting Guide, 2024). If the best-fit value was well centered (i.e., the 95% CI was symmetrical or close to it), the parameterizations of the model were regarded appropriate.

When setting the way in which the software checks how well the experimental data define the model, both available options were used, sc. “identifying ambiguous fits” and “identifying unstable parameters” (Curve Fitting Guide, 2024). The reason for this was that more estimates of the distorting concentration could be obtained with “identifying ambiguous fits” (an option dealing with the extent of parameter intertwining) than with “identifying unstable parameters” (the default option). When RRM was implemented with “identifying unstable parameters”, some estimates (labelled as “ambiguous” by the former option) were missing, while the rest had practically the same values and 95% CIs.

For almost all cases, the default option for computing 95% CIs was “asymmetrical” (Curve Fitting Guide, 2024). Where not (during defining the models to fit), the “asymmetrical” option was chosen. For every setting not addressed above, the default option was used.

## 3 Results

### 3.1 The influence of the form of the fitted equation (regarding its complexity and the expression of the distorting concentration)

Upon individual fitting and two-model global regression for the distorted E/c curves, all equations provided practically the same estimates (not affected by the ordinary or robust way of fitting). Upon individual regression for the intact E/c curves, the estimates, while being somewhat different for the different equations, showed small, close-to-zero or zero values (as expected). For the sake of simplification, only data provided by Eqs 7, 8 have been presented throughout this paper (cf. Table 2, 3).

**TABLE 2:** The logc_x_ best-fit values (provided by the Eq 8), with 95% confidence intervals (95% CI) and antilog values (c_x_, converted to nmol/L, as estimates, highlighted in bold), obtained with the receptorial responsiveness method (RRM) from data of six groups (see column headers) of a previous *ex vivo* study (Szabo et al., 2019a), using three fitting ways combined with two another fitting options (see row headers). na: not applicable; nM: nmol/L; CPA: *N* ^*6*^-cyclopentyladenosine; NECA: 5′-(N-ethylcarboxamido)adenosine; CHA: *N* ^*6*^-cyclohexyladenosine

			CPA E/c curves		NECA E/c curves			CHA E/c curves	
			Intact	Distorted (100 nM)	Intact		Distorted (100 nM)	Intact	Distorted (300 nM)
**Individual**	**ordinary**	logc_x_	−19107	−6.88	−8.92	−6.87		−52574	−6.45
		95% CI	*very wide*	−6.93–−6.83	?–−8.32	−6.92–−6.83		*very wide*	−6.55–−6.36
		**c**_**x**_ **(nM)**	**≈ 0**	**131.4**	**1.2**	**133.6**		**≈ 0**	**352.7**
	**robust**	logc_x_	−19107	−6.9	−8.64	−6.88		−52574	−6.47
		**c**_**x**_ **(nM)**	**≈ 0**	**125.9**	**2.3**	**131.4**		**≈ 0**	**335.8**
**Global, 1 model**	**ordinary**	logc_x_	−7201	−6.84	−35267	−6.77		−1.17∙10^10^	−6.39
		95% CI	*very wide*	*very wide*	*very wide*	*very wide*		*very wide*	*very wide*
		**c**_**x**_ **(nM)**	**≈ 0**	**145.9**	**≈ 0**	**170**		**≈ 0**	**403.7**
	**robust**	logc_x_	−7209	−6.85	−35267	−6.82		−1.17∙10^10^	−6.36
		**c**_**x**_ **(nM)**	**≈ 0**	**142.6**	**≈ 0**	**153.3**		**≈ 0**	**439**
**Global, 2 models**	**ordinary**	logc_x_	na	−6.84	na	−6.77		na	−6.39
		95% CI		−6.97–−6.7		−6.87–−6.67			−6.62–−6.17
		**c**_**x**_ **(nM)**		**145.9**		**170**			**403.7**
	**robust**	logc_x_	na	−6.85	na	−6.82		na	−6.36
		**c**_**x**_ **(nM)**		**142.7**		**153.2**			**439**

**TABLE 3:** The c_x_ best-fit values (provided by the Eq. 7) converted to nmol/L (as estimates, highlighted in bold), with 95% confidence intervals (95% CI), obtained with the receptorial responsiveness method (RRM) from data of six groups (see column headers) of a previous *ex vivo* study (Szabo et al., 2019a), using three fitting ways combined with two another fitting options (see row headers). na: not applicable; nM: nmol/L; CPA: *N* ^*6*^-cyclopentyladenosine; NECA: 5′-(N-ethylcarboxamido)adenosine; CHA: *N* ^*6*^-cyclohexyladenosine

			CPA E/c curves		NECA E/c curves			CHA E/c curves	
			Intact	Distorted (100 nM)	Intact		Distorted (100 nM)	Intact	Distorted (300 nM)
**Individual**	**ordinary**	**c**_**x**_ **(nM)**	**7.18∙10** ^**−**^ **^17^**	**131.4**	**1.23**	**133.6**		**2.57∙10** ^**−**^ **^16^**	**352.7**
		95% CI (nM)	?	116.9	?	119.5		?	281.8
			0.91	147.2	4.83	148.6		6.72	436.5
	**robust**	**c**_**x**_ **(nM)**	**6.22∙10** ^**−**^ **^17^**	**125.9**	**2.27**	**131.4**		**8.18∙10** ^**−**^ **^17^**	**335.8**
**Global, 1 model**	**ordinary**	**c**_**x**_ **(nM)**	**6.08**	**139.7**	**20.07**	**211.9**		**24.28**	**357.4**
		95% CI (nM)	*very wide*	*very wide*	*very wide*	143.9		*very wide*	*very wide*
						?			
	**robust**	**c**_**x**_ **(nM)**	**3.53∙10** ^**−**^ **^11^**	**143**	**29.5**	**196.1**		**4.24∙10** ^**−**^ **^11^**	**451.2**
**Global, 2 models**	**ordinary**	**c**_**x**_ **(nM)**	na	**145.9**	na	**170**		na	**403.7**
		95% CI (nM)		107.8		136.4			238.7
				199.3		211.9			682.6
	**robust**	**c**_**x**_ **(nM)**	na	**142.7**	na	**153.2**		na	**439**

Upon one-model global regression, however, the fitting of the Eq 6 failed completely, it provided no or obviously wrong estimates (accompanied by error messages from the curve fitting software). The estimates yielded by the Eqs 7, 8 differed, although to a moderate extent (regardless the use of ordinary or robust fitting). The estimates for the intact E/c curves provided by the Eq 8 were clearly better (i.e., closer to zero) than those yielded by the Eq 7 (cf. Table 2, 3).

According to the 95% CIs as well as 95% confidence and prediction bands (being accessible only with ordinary fitting), there was no considerable difference in the precision between the models containing c_x_ and logc_x_ (if the fitting was possible) (cf. Table 2, 3, furthermore the adjacent left and right panels of Figures 1–3). As a surprising exception, during one-model global fitting to NECA E/c curves, confidence and prediction bands were only displayable (i.e., only then were they not undisplayably poor) when using the Eq 7 (Figure 2).

Summarizing, in general, the Eq 8 was better than the Eq 7, while the Eq 7 was better than the Eq 6. So, three conclusions could be drawn: **1**) the use of c_x_ (instead of logc_x_) in the model of RRM did not improve either accuracy or precision of the estimation; **2**) the simpler model of RRM worked more reliably than the more complex, although algebraically equivalent model; **3**) avoiding division by c_x_ was of no benefit for the estimation (even in the case of the intact E/c curves).

### 3.2 The influence of the number and use of models

Accuracy was the best in the case of individual fitting, which was followed by the other options with only moderate differences. When using the model of RRM with logc_x_, accuracy was not affected at all by the fact whether one- or two-model global fitting was chosen. Accuracy was slightly influenced by whether ordinary or robust regression was carried out, and it was moderately influenced by whether c_x_ or logc_x_ was in the fitted model (this latter observation was especially true in the case of one-model global fitting). All estimates for the intact E/c curves showed small values, especially when using logc_x_ to fit (Table 2, 3).

According to the 95% CIs, precision was the best upon individual fitting, but only when the distorted E/c curves were assessed. Precision was acceptable when using two-model global fitting (always), whereas it was unquantifiably poor in the case of one-model global regression (always) and individual fitting (when assessing intact E/c curves) (Table 2, 3). For the one-model global regression, the cause of the ambiguous fit was that c_x_ and logc_x_ in the Eqs 7, 8, respectively, were highly intertwined (i.e., showed strong correlation) with the other parameters. The use of c_x_ (in the Eq 7) substantially increased the correlation even between parameters other than c_x_. Moreover, the use of the Eq 6 (also containing c_x_) prevented the successful curve fitting during one-model global regression.

The results provided by the 95% confidence and prediction bands were supportive of those yielded by the 95% CIs, with one exception. Namely, the 95% confidence bands for the intact E/c curves, obtained with individual fitting, seem to be surprisingly narrow, when considering the very wide 95% CIs for the corresponding best-fit values (Table 2, 3; Figure 1). This phenomenon was especially conspicuous when using the model with logc_x_ (Table 2; Figure 1, left panels). This might be due to that the best-fit values involved (both c_x_ and logc_x_) were very small (as expected in the case of intact E/c curves), so even a relatively wide range around these c_x_ and logc_x_ values could appear to be narrow. Therefore, these narrow confidence bands might be misleading, while the much wider 95% prediction bands, holding information about the uncertainty of the E/c curve data in addition to the uncertainty of the curve fitting, could be considered more illustrative (Figure 1).

Overall, the two-model global regression could be regarded as the most precise, but the individual regression was not far behind, especially if considering that the estimates for the intact E/c curves served only as an additional control (Table 2, 3; Figures 1–3).

Regarding the convenience of use, the global way of regression was undoubtedly superior to the individual one. Specifically, the one-model global fitting was the most convenient (because it did not require creating a multiline model in the software; see Appendix A). In terms of convenience, it had no relevance whether c_x_ or logc_x_ was fitted and in which equation, furthermore whether ordinary or robust regression was performed.

In addition, every best-fit value, for which 95% CI could be computed, was well centered in it, indicating the proper parametrizations of the fitted models.

### 3.3 The influence of the number of distorted E/c curves fitted at once

It is worth noting in advance that, upon global regression, the assessment of an E/c curve family offers an extra choice for the implementation of RRM: in addition to all-at-once fitting, it is possible to fit in a pairwise manner (that increases the impact of the intact E/c curve on the results). For simplicity, only Eqs 2, 8 were used to evaluate the E/c curve families.

In terms of accuracy, all modes of regression provided similarly good estimates for the greater two of the three distorting concentrations (within a given E/c curve family). However, the estimates for the smallest distorting concentration were acceptable only when individual regression or global regression with pairwise fitting was performed. The estimates for the intact E/c curves (where relevant) were close to zero (Table 4, 5).

**TABLE 4:** The logc_x_ best-fit values (yielded by the Eq 8), with 95% confidence intervals (95% CI) and antilog values (c_x_, converted to nmol/L, as estimates, highlighted in bold), obtained with the receptorial responsiveness method (RRM) from data of four groups (see column headers) of a previous *ex vivo* study (Gesztelyi et al., 2004), using three fitting ways combined with two another fitting manners (see row headers). In the case of global regression, curve fitting was carried out by pairing each Distorted group with the related Intact one (“pairwise technique”). na: not applicable; nM: nmol/L; CPA: *N* ^*6*^-cyclopentyladenosine

Fit to curve pairs			CPA E/c curves					
(+indiv)			Intact	Distorted 3 nM	Intact	Distorted 30 nM	Intact	Distorted 100 nM
**Individual**	**ordinary**	logc_x_	−14.66	−8.1	-	−7.58	-	−7.02
		95% CI	*very wide*	−8.2–−8.01	-	−7.62–−7.55	-	−7.09–−6.95
		**c**_**x**_ **(nM)**	**2.2∙10** ^**−**^ **^6^**	**8.02**	**-**	**26.06**	**-**	**95.5**
	**robust**	logc_x_	−20375	−8.06	-	−7.59	-	−6.99
		**c**_**x**_ **(nM)**	**≈ 0**	**8.66**	**-**	**25.46**	**-**	**101.4**
**Global, 1 model**	**ordinary**	logc_x_	−7237	−8.08	−7.04∙10^8^	−7.54	−1.52∙10^7^	−6.8
		95% CI	*very wide*	*very wide*	*very wide*	*very wide*	*very wide*	*very wide*
		**c**_**x**_ **(nM)**	**≈ 0**	**8.37**	**≈ 0**	**28.95**	**≈ 0**	**159.2**
	**robust**	logc_x_	−7237	−8.07	−7.04∙10^8^	−7.53	−1.52∙10^7^	−6.8
		**c**_**x**_ **(nM)**	**≈ 0**	**8.46**	**≈ 0**	**29.83**	**≈ 0**	**158.3**
**Global, 2 models**	**ordinary**	logc_x_	na	−8.08	na	−7.54	na	−6.8
		95% CI		−8.25–−7.94		−7.62–−7.46		−6.93–−6.65
		**c**_**x**_ **(nM)**		**8.37**		**28.95**		**159.2**
	**robust**	logc_x_	na	−8.07	na	−7.53	na	−6.8
		**c**_**x**_ **(nM)**		**8.46**		**29.83**		**158.3**

**TABLE 5:** The logc_x_ best-fit values (provided by the Eq 8), with 95% confidence intervals (95% CI) and antilog values (c_x_, converted to nmol/L, as estimates, highlighted in bold), obtained with the receptorial responsiveness method (RRM) from data of four groups (see column headers) of a previous *ex vivo* study (Gesztelyi et al., 2004), using the two manners of global regression combined with two another fitting options (see row headers). Curve fitting was carried out simultaneously to all groups (“all-at-once technique”). na: not applicable; nM: nmol/L; CPA: *N* ^*6*^-cyclopentyladenosine

Fit to all curves			CPA E/c curves			
			Intact	Distorted 3 nM	Distorted 30 nM	Distorted 100 nM
**Global, 1 model**	**ordinary**	logc_x_	−1122	−198.9	−7.51	−6.85
		95% CI	*very wide*	*very wide*	*very wide*	*very wide*
		**c**_**x**_ **(nM)**	**≈ 0**	**1.14∙10** ^**−**^ **^190^**	**30.94**	**142.1**
	**robust**	logc_x_	−1122	−198.9	−7.5	−6.86
		**c**_**x**_ **(nM)**	**≈ 0**	**1.14∙10** ^**−**^ **^190^**	**31.47**	**138.2**
**Global, 2 models**	**ordinary**	logc_x_	na	−116434	−7.51	−6.85
		95% CI		*very wide*	*very wide*	*very wide*
		**c**_**x**_ **(nM)**		**≈ 0**	**30.94**	**142.1**
	**robust**	logc_x_	na	−116434	−7.5	−6.86
		**c**_**x**_ **(nM)**		**≈ 0**	**31.47**	**138.3**

**FIGURE 4:**
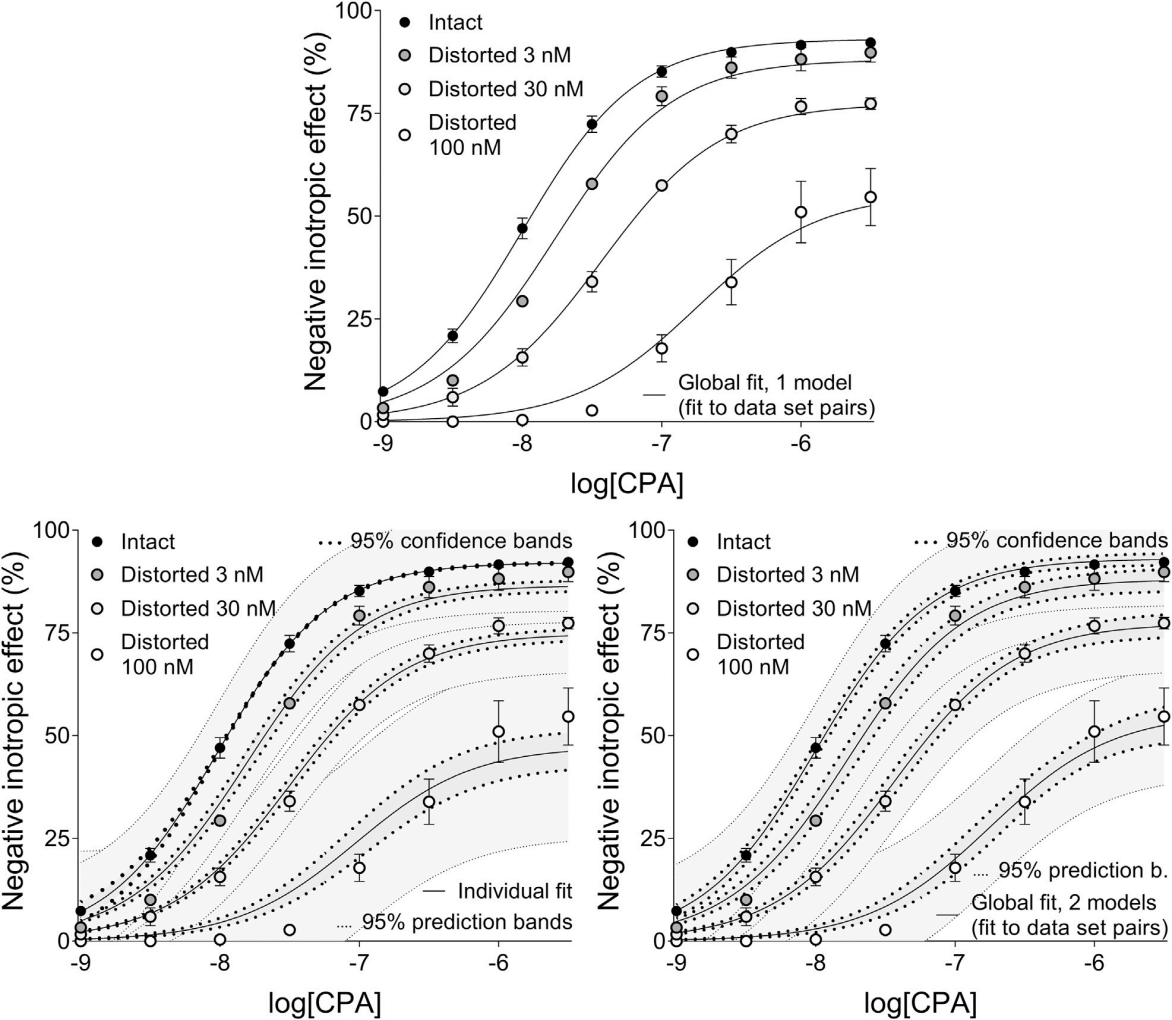
The implementation of RRM with one-model global regression (panel above), individual regression (left panel below) and two-model global regression (right panel below) combined with ordinary least-squares fitting, using exclusively the common logarithm of the concentration to be determined (logc_x_) as a parameter. The procedure was performed on concentration-effect (E/c) curve families, each consisting of one “Intact” and three “Distorted” E/c curves, obtained from an earlier *ex vivo* study (Gesztelyi et al., 2004). Both ways of global regression were performed in pairwise manner (i.e., only two E/c curves at once, one of which was always the “Intact” E/c curve). The x-axis shows the common logarithm of the molar concentration of CPA, a synthetic A_1_ adenosine receptor full agonist, administered for constructing the E/c curves. The y-axis indicates the effect (the percentage decrease in the initial contractile force of isolated, paced guinea pig left atria). The “Intact” E/c curves (black symbols) were generated conventionally, while the “Distorted” E/c curves (symbols filled with a color from dark gray to white) were constructed in the presence of a single, disregarded surplus CPA concentration (which latter increased as the color of the symbol lightened). The symbols indicate the responses to CPA averaged within the groups (±SEM). The continuous lines show the best-fit curves of the fitted Eqs 2, 8 (chosen according to the way of fitting and to the type of the E/c curve). The thick dotted lines indicate the 95% confidence bands, while the thin dotted lines denote the 95% prediction bands (if any). RRM: receptorial responsiveness method; CPA: *N* ^*6*^-cyclopentyladenosine.

Precision achieved with global regression carried out with pairwise technique was similar in all respects to that described in the previous subsection for the Eq 8 (Table 4, 5; Figure 4). Upon global regression performed in all-at-once manner, however, precision was unquantifiably poor (irrespective of the number of models). Thus, the two-model global regression, implemented with pairwise fitting, was considered the most precise, but it was closely followed by the individual regression.

Regarding manageability, the one-model global regression performed with all-at-once fitting was the most convenient to use, it was followed by the one-model global regression with pairwise fitting, then the two-model global regression with all-at-once fitting, and then the two-model global regression with pairwise fitting. Eventually, the most complicated way to perform RRM was the individual regression in this case as well.

Every best-fit value was well centered in its 95% CI (if any), thus the parametrizations of the models were found to be appropriate here as well.

The main results of the study have been summarized in Table 6.

**TABLE 6:** Comparison of the results of the receptorial responsiveness method (RRM) performed with individual or global fitting and all-at-once or pairwise techniques. These conclusions have been drawn for the model containing logc_x_ (generally found to be better than models including c_x_), but roughly the same is true for the models with c_x_.

	Individual	Global			
	(Local)	Fit to all curves		Fit to curve pairs	
		One model	Two models	One model	Two models
**Accuracy**	very good	poor/good	poor/good	good	good
**Precision**	good	poor	poor	poor	very good
**Manageability**	arduous	very easy	easy	easy	complicated

## 4 Discussion

In the present study, different implementations of RRM were tested by estimating known concentrations of three stable adenosine receptor agonists, NECA, CPA and CHA, in the vicinity of the A_1_ adenosine receptors located in the guinea pig atrial myocardium.

RRM is a simple (i.e., non-multiple) nonlinear regression-based procedure with a unique model containing two variables (c or its logarithm logc, and E′) plus at least one variable parameter (c_x_ or its logarithm logc_x_). The c is the concentration of an agonist that is administered to generate an E/c curve, while E’ is the effect (related to the E/c curve) that is partly (but not completely) evoked by c in a biological system. As for the obligate variable parameter (c_x_ or logc_x_), its role is, in the broadest sense, to quantify something, which (before the generation of the E/c curve) has decreased the responsiveness of the given biological system. This quantification is made by RRM with the concentration of the agonist used for the E/c curve that is capable of producing the same reduction in the responsiveness as the original evoking factor. In a simple case, the “something” to be quantified is a single, constant concentration of an agonist (called “distorting agonist”, the quantification of which is thus the goal of RRM). In turn, in the simplest case, the agonist used for the E/c curve and the distorting agonist are the same, a case when RRM directly estimates the concentration of the distorting agonist (as c_x_ or as logc_x_) (Gesztelyi et al., 2004; Grenczer et al., 2010a; 2010b).

Although RRM deals with an inherently linear issue (see the Eq. 3), the relationship between a concentration (or dose) and a biological effect is typically nonlinear. Thus, we arranged the model of RRM (intended for curve fitting) to be nonlinear *via* combining the basic equation of RRM with a nonlinear receptor function model (being generally the Hill equation). This way, the model of RRM became suitable to evaluate raw (or minimally transformed) E/c data (Gesztelyi et al., 2004; Grenczer et al., 2010a). This yields more reliable results because the more the data to be fitted are transformed, the greater the risk that the biological variability and measurement errors in the raw data will significantly bias the results (Motulsky and Christopoulos, 2004).

Beyond the simple concentration estimation (such as the determination of the interstitial accumulation of the endogenous adenosine during nucleoside transport blockade: Karsai et al., 2006; 2007), RRM also meets other challenges. RRM is suitable to correct E/c curves of adenosine receptor agonists for the distortion caused by a change in the endogenous adenosine level. Accordingly, RRM helped to uncover the slight positive inotropic effect of EHNA that resulted probably in part from phosphodiesterase type 2 (PDE2) inhibition and partly from an unclear mechanism characteristic of potent adenosine deaminase inhibitors (Gesztelyi et al., 2003; Kemeny-Beke et al., 2007; Pak et al., 2015). The use of RRM also enabled the discovery of a hitherto unknown property of FSCPX, a well-established irreversible A_1_ adenosine receptor antagonist, namely, the ability to decrease the interstitial adenosine concentration (*via* an unknown mechanism), in a series of *in silico* (Zsuga et al., 2017; Szabo et al., 2019b) and *ex vivo* investigations (Erdei et al., 2018; Viczjan et al., 2021). Furthermore, RRM contributed to the assessment of the A_1_ adenosine receptor reserve for the direct negative inotropic effect of adenosine, an agonist difficult to quantify *ex vivo* (and *in vivo*) due to its short half-life (Kiss et al., 2013; Pak et al., 2014; Zsuga et al., 2017). In addition, RRM was used to decide which one of two possible actions of cannabidiol, a widely used phytocannabinoid, can be responsible for the enhancement of the adenosinergic activity (this was found to be the nucleoside transport blockade rather than direct A_1_ adenosine receptor agonism) (Viczjan et al., 2022).

The basic equation of RRM (Eq 3) describes the link between a distorted effect (measured in a system with decreased responsiveness) and the corresponding intact effect (determined in the same system in its intact state). Encompassing this relationship, the model of RRM (Eqs 4–8) connects E’, the distorted effect, with c, the known concentration of an agonist (or with logc, its logarithm) and with the cause of the decreased responsiveness that is expressed as c_x_, a distorting concentration (or logc_x_, its logarithm). Importantly, for simplicity, c_x_ is attributed to the same agonist as c. The model of RRM incorporates a quantitative model of the receptor function as well and thereby uses its parameters (herein and mostly it is the Hill model: Eq 2). It should be noted that, from a theoretical point of view, the model of RRM can be considered oversimplified when applied to data resulted from the co-action of two different agonists. This circumstance might be responsible for some of the shortcomings of this method (Grenczer et al., 2010a; Grenczer et al., 2010b).

As input for RRM, at least two (types of) E/c curves are needed: an “intact” E/c curve that displays the effect of the agonist used to generate the E/c curves in the system possessing its intact responsiveness, and a “distorted” one that shows the effect of this agonist in the system with decreased response capacity. The distorted E/c curve should be fitted to the model of RRM using some information obtained from the related intact E/c curve (Gesztelyi et al., 2004; Grenczer et al., 2010a; Szabo et al., 2019a). The way this information is transferred is what distinguishes the individual (local) regression, the one-model global regression and the two-model global regression (as three options for implementing RRM). In the present investigation, one of our goals was to find out which of these options is optimal for RRM. In addition, the two-model global fitting was also used as an alternative tool to clarify the significance of logc_x_ in the model of RRM (in terms of fitting the intact E/c curves, see below).

A surprising property of regression (at least for non-mathematicians) is that the fitting of different but algebraically equivalent expressions can lead to somewhat different results (Young, 2017). Consequently, another goal of this study was to explore whether the simplification of the algebraic form of the RRM’s model can improve the outcome of RRM. Because the simplification used here included a division by c_x_, a parameter that should otherwise be allowed to be zero, results obtained this way could reflect the consequences of both the simplification and the division by c_x_, which latter operation theoretically excludes the possibility of c_x_ to be zero.

Not independently from the fact that different but equivalent equations can provide different results, it was recommended that a parameter following log-normal (Galton) distribution (e.g., all kinds of concentration) should be used as a common logarithm in models intended for curve fitting, in order to obtain a symmetrical CI (Motulsky and Christopoulos, 2004). In the model of RRM, the distorting concentration can also be expressed as either a logarithm (logc_x_) or a numerus (c_x_), options that may affect the results of the estimation. However, as the software used for curve fitting in the present study can handle asymmetrical CIs as well, it seemed reasonable to revisit, which form of the distorting concentration is better for fitting. Therefore, in the present study, three different but algebraically equivalent models of RRM (Eqs 6-8) were used to explore the influence of the following three interventions on the accuracy and precision of RRM: **1**) simplification of the model of RRM; **2**) division by c_x_ in the RRM’s model (inseparable from the simplification); and **3**) changing the form of the distorting concentration (c_x_ or logc_x_) in the RRM’s model.

The regression ways mentioned so far can be combined with two additional fitting options, i.e., ordinary and robust regression, to consider the distribution of the scatter of data points around the best-fit curve. This distribution can be closer to either Gaussian or Lorentzian distribution (the two extremes of the t distribution regarding the number of degrees of freedom), which conditions require ordinary or robust regression, respectively. It should be noted that the use of robust regression limits the usefulness of the estimation as it prevents the determination of precision (Curve Fitting Guide, 2024).

Furthermore, if more than one distorted E/c curve belongs to one intact E/c curve, and global regression is chosen, there will be two possibilities to perform RRM: fitting all related E/c curves at once or fitting them pairwise (i.e., each distorted E/c curve separately with the intact E/c curve). These two options may also lead to different results to be addressed.

In the present study, accuracy of RRM has generally been found to be acceptable for all kinds of regression used herein (Table 2–5). Nevertheless, there were three noteworthy exceptions: **1**) the one-model global fitting of the Eq 6 (the non-simplified model of RRM with c_x_); **2**) the one-model global fitting for the intact E/c curves with the Eq 7 (the simplified model with c_x_) (Table 3); and **3**) the global fitting performed in all-at-once manner to determine the smallest distorting concentration (obtained from Gesztelyi et al., 2004) (Table 5). From these observations, three major conclusions can be drawn: **1**) in the model of RRM, the use of c_x_ is rather a disadvantage than an advantage; **2**) the algebraically simplified model is better for RRM (even despite the theoretical concern of dividing by c_x_, a parameter that should be allowed to be zero); and **3**) a small distorting concentration is a challenge for RRM, especially if it is determined by fitting an E/c curve family in all-at-once manner. These conclusions are detailed as follows.

Ad **1**): Rewriting the RRM’s model to replace logc_x_ with c_x_ did not improve either accuracy or precision of the estimation. On the contrary, in some cases, the use of c_x_ worsened precision by increasing the correlation between some parameters, moreover, the combination of the Eq 6 (the original complex model entirely allowing c_x_ to be zero) with the one-model global fitting made the determination impossible. This finding has refuted our previous assumption about the poor performance of the one-model global regression implemented with a model containing logc_x_ (Szabo et al., 2019a). So, foibles of RRM in any case do not stem from the fit of logc_x_ that would hinder the correct determination of the zero value of c_x_ for the intact E/c curves. This conclusion has been confirmed by another finding of the present study. Namely, the two-model global regression (with a model containing either logc_x_ or c_x_) did not improve the accuracy of RRM in comparison to either the one-model global fitting or the individual regression (Table 2, 3), although it has proven to be the most precise (Table 2, 3; Figures 1–3).

Ad **2**): The problems in the estimation observed in some cases may be the results of the relative complexity of the model of RRM (even for the algebraically simplified model expressed by the Eq 8). The greater the complexity of a regression model, the greater the degree of correlation that can occur between the parameters of the model (Motulsky and Christopoulos, 2004; Curve Fitting Guide, 2024). Thus, for RRM, a model as simple as possible should be used (Table 1). In addition, in cases when the agonist used for the E/c curves and the distorting agonist are different, the causal role of the theoretical oversimplification included in the model of RRM may also be significant in terms of accuracy as well as precision (Grenczer et al., 2010a; 2010b).

Ad **3**): This conclusion is consistent with our earlier *in silico* finding that too small and too large concentrations might be estimated with substantial inaccuracy by means of RRM (Grenczer et al., 2010a; 2010b). (A distorting concentration can be regarded small or large if its effect, namely, E_x_ in the Eq 3, is small or large relative to the maximal effect of the agonist used for the E/c curves, i.e., E_max_ in Eqs 4–8, respectively.) However, for E/c curve families, accuracy (when a small concentration was to be determined) and precision (in every case) of the global regression could be substantially improved by fitting the appropriate E/c curves pairwise (cf. Table 4, 5). Thus, despite the acceptable estimation for most cases and the good usability, we do not recommend fitting globally to more than two E/c curves at once (for RRM). In line with this, unless otherwise indicated, we discuss those results of global regression that were obtained with pairwise fitting technique.

As for the further fitting options, in terms of accuracy, the individual fitting proved to be the best, followed closely by the global regression (when fitting the model with logc_x_, irrespective of the number of models). In turn, regarding precision (i.e., the reliability of the estimates), the two-model global fitting was the best, followed closely by the individual regression and, from afar, by the one-model global fitting (Table 2–5). As regards manageability, the one-model global regression was the best, followed by the moderately complicated two-model global fitting, and then by the most complicated individual regression (Table 6).

The poor precision of the one-model global regression has spectacularly been indicated by the fact that (of course upon ordinary fitting) neither confidence intervals nor confidence and prediction bands could be obtained for the results (an interesting exception was seen for the NECA E/c curves when fitting the Eq 7) (Figure 2). The source of this uncertainty may be that the parameter estimating the distorting concentration (c_x_ or logc_x_) was greatly dependent on the other parameters when fitting the intact E/c curves. The other occasion, when similarly high level of parameter correlation occurred, was the attempt to determine the smallest distorting concentration by fitting simultaneously more than two E/c curves (affecting both types of global regression).

In favor of the oldest way to perform RRM, it should be noted that the individual regression imposes the fewest requirements for the curve fitting software used. Nevertheless, there are nowadays much software with great capabilities to choose from.

Additionally, most differences among the various implementations of RRM have been found in terms of precision and convenience of use. Accordingly, the choice between ordinary or robust regression had little impact in this study, as the robust regression prevented calculating confidence intervals for the best-fit values as well as confidence and prediction bands for the best-fit curves. Besides, the robust regression did not affect the convenience (manageability) of curve fitting at all (Table 2–5).

As a final conclusion: the use of RRM is recommended with **1**) the simplified model containing logc_x_; **2**) two-model global fitting (pairwise, if appropriate), or possibly individual fitting; **3**) ordinary least-squares regression, or possibly robust regression.

## Data Availability

The original contributions presented in the study are included in the article, further inquiries can be directed to the corresponding author.

## Appendix

In this section, the equations have been presented as they were written into the curve fitting software. Consistently, the independent and dependent variables are X and Y here (while elsewhere they are logc and E or E’, respectively).

As Hill model, the software’s built-in equation “sigmoidal dose-response (variable slope)”, with a “bottom” parameter constrained to zero (Curve Fitting Guide, 2024), was used (see the Eq. 2):

**Y = Emax/(1 + 10 ^ (n*(logEC50-X)))**

Eqs 6 and 7 and 8, respectively, prepared for the individual fitting and one-model global fitting:

**Y = 100–100*(100-Emax*(cx+10^X)^n/((cx+10^X)^n+10^(n*logEC50)))/(100-Emax*cx^n/(cx^n+10^(n*logEC50)))**

and

**Y = 100–100*(100-Emax/(1 + 10^(n*(logEC50-log (cx+10^X)))))/(100-Emax/(1 + 10^(n*(logEC50-log (cx)))))**

and

**Y = 100–100*(100-Emax/(1 + 10^(n*(logEC50-log (10^logcx+10^X)))))/(100-Emax/(1 + 10^(n*(logEC50-logcx))))**

Multiline models involving Eq. 2 combined with Eqs 6 and 7 and 8, respectively, prepared for the two-model global fitting:

**Hill = Emax/(1 + 10^(n*(logEC50-X)))**

**RRM = 100–100*(100-Emax*(cx+10^X)^n/((cx+10^X)^n+10^(n*logEC50)))/(100-Emax*cx^n/(cx^n+10^(n*logEC50)))**

**<A>Y=Hill**

**<∼A > Y = RRM**

and

**Hill = Emax/(1 + 10^(n*(logEC50-X)))**

**RRM = 100–100*(100-Emax/(1 + 10^(n*(logEC50-log (cx+10^X)))))/(100-Emax/(1 + 10^(n*(logEC50-log (cx)))))**

**<A>Y=Hill**

**<∼A > Y = RRM**

and

**Hill = Emax/(1 + 10^(n*(logEC50-X)))**

**RRM = 100–100*(100-Emax/(1 + 10^(n*(logEC50-log (10^logcx+10^X)))))/(100-Emax/(1 + 10^(n*(logEC50-logcx))))**

**<A>Y=Hill**

**<∼A > Y = RRM**

(For more information, see “fitting different models to different data sets” in: Curve Fitting Guide, 2024.)
